# Macrophage Activation Markers, Soluble CD163 and Mannose Receptor, in Liver Fibrosis

**DOI:** 10.3389/fmed.2020.615599

**Published:** 2021-01-08

**Authors:** Rasmus Hvidbjerg Gantzel, Mikkel Breinholt Kjær, Tea Lund Laursen, Konstantin Kazankov, Jacob George, Holger Jon Møller, Henning Grønbæk

**Affiliations:** ^1^Department of Hepatology and Gastroenterology, Aarhus University Hospital, Aarhus, Denmark; ^2^Institute for Liver and Digestive Health, University College London, London, United Kingdom; ^3^Storr Liver Centre, Westmead Institute for Medical Research, University of Sydney and Westmead Hospital, Westmead, NSW, Australia; ^4^Department of Clinical Biochemistry, Aarhus University Hospital, Aarhus, Denmark

**Keywords:** liver, fibrosis, macrophages, sCD163, mannose receptor, metabolic associated fatty liver disease, hepatitis B virus, hepatitis C virus

## Abstract

Macrophages are essential components of the human host immune system, which upon activation facilitates a broad pallet of immunomodulatory events including release of pro- or anti-inflammatory cytokines and chemokines, restoration of immune homeostasis and/or wound healing. Moreover, some macrophage phenotypes are crucially involved in fibrogenesis through stimulation of myofibroblasts, while others promote fibrolysis. During the last decades, the role of resident liver macrophages viz. Kupffer cells and recruited monocytes/macrophages in acute and chronic liver diseases has gained interest and been extensively investigated. Specifically, the scavenger receptors CD163 and mannose receptor (CD206), expressed by macrophages, are of utmost interest since activation by various stimuli induce their shedding to the circulation. Thus, quantifying concentrations of these soluble biomarkers may be of promising clinical relevance in estimating the severity of inflammation and fibrosis and to predict outcomes such as survival. Here, we review the existing literature on soluble CD163 and soluble mannose receptor in liver diseases with a particular focus on their relationship to hepatic fibrosis in metabolic associated fatty liver disease, as well as in chronic hepatitis B and C.

## Introduction

The progression course from low grade hepatic inflammation and early stage fibrosis to manifest cirrhosis differs depending on disease etiology but also between subgroups within the same disease (1). With established cirrhosis follow substantial reductions in quality of life and survival, especially when decompensation develops with variceal bleeding, hepatic encephalopathy, ascites and/or hepatorenal syndrome or the development of liver cancer (2–4).

The pathomechanisms orchestrating disease progression and maintenance of inflammation and fibrosis are regulated through a complex interplay between immune cells. Resident liver macrophages viz. Kupffer cells along with recruited monocytes/macrophages are essential in the development and progression of liver diseases (5). Macrophages are key players of human innate immunity displaying an extensive array of membrane receptors, which mediate crucial immunomodulatory responses upon macrophage activation with both pro- and anti-inflammatory effects (6, 7). We review the value of two biomarkers of macrophage activation in liver diseases and their relationship to the degree of fibrosis, ultimately concluding with the potential clinical utility of these biomarkers. We briefly comment on macrophage-related therapeutic options.

## Macrophages and Liver Fibrosis

Kupffer cells and recruited macrophages possess the ability to exert omnipotent immunomodulation, evident by both pro-fibrotic and anti-fibrotic effects in the liver (8). Traditionally, macrophages have been divided in two subgroups with either a “pro-inflammatory” M1 or “immune-regulatory” M2 phenotype. However, this dichotomous classification is a too simplified division of a highly heterogeneous group of differently activated and functioning immune cells as recently affirmed by advanced molecular sequencing techniques (8–11).

**Figure 1:**
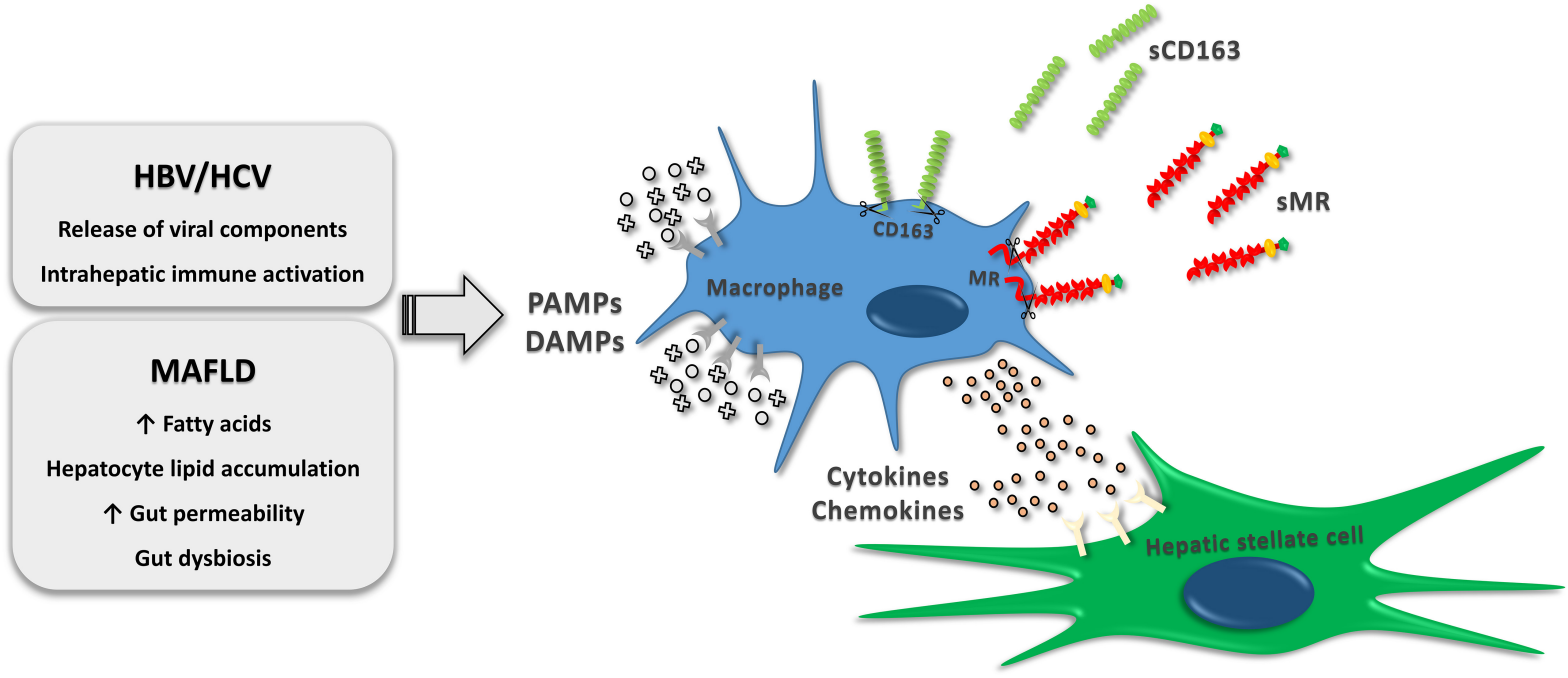
Macrophage activation by immunomodulatory events occurring in MAFLD and chronic HBV or HCV infections. The activation increase receptor shedding, which results in release of soluble CD163 and soluble MR to the circulation. Furthermore, cytokines and chemokines released by resident and recruited liver macrophages stimulate hepatic stellate cell differentiation into myofibroblasts consequently causing synthesis of extracellular matrix components. HBV, hepatitis B virus; HCV, hepatitis C virus; MAFLD, metabolic associated fatty liver disease; PAMPs, pathogen-associated molecular patterns; DAMPs, damage-associated molecular patterns; sCD163, soluble cluster of differentiation 163; sMR, soluble mannose receptor.

Pathogens reaching the hepatic immune environment initiate a pro-inflammatory response with Kupffer cells as essential partakers and a major source of inflammatory signaling molecules (12). Macrophage activation by pathogen-associated molecular patterns (PAMPs) through pattern-recognizing receptors (PRRs) including Toll-like-receptors is of utmost relevance in the immunological processes during chronic inflammation. Similarly, macrophages may be activated by damage-associated molecular patterns (DAMPs) (Figure 1) (13). The cytokines interleukin-12 (IL-12) and interferon-γ (IFN-γ) are known to initiate a pro-inflammatory response in macrophages, resulting in the release of tumor necrosis factor (TNF), IL-1β, IL-6, IL-12 and reactive oxygen species (7–9). Furthermore, the presence of a IFNL3-IFNL4 haplotype resulting in production of IFN-γ3 is recognized as a promoter of hepatic inflammation and fibrosis progression (14). Through the release of pro-inflammatory cytokines and chemokines, macrophages interplay with other immune cells including T cells, B cells, natural killer T cells and neutrophils (8, 15). Conversely, the release of modulatory cytokines such as IL-10, transforming growth factor β (TGF-β), IL-4 and IL-13, induced by adaptive and innate signals, is of key importance in dampening inflammation and promoting wound healing (7–9, 16). In this view, TGF-β potently stimulates hepatic stellate cell differentiation into myofibroblasts (17) responsible for fibrogenesis during bouts of hepatic inflammation. Myofibroblasts undergo persistent proliferation contributing to disease progression by synthesis of extracellular matrix components and potentiation of ongoing inflammation with the release of chemokines, cytokines and fibrogenic mediators (18–20). Upon dampening of liver injury, histoarchitecture restorative events become dominating with deactivation of myofibroblasts by apoptosis or phenotypic switch and activation of protective or restorative macrophages, which promote fibrosis resolution and tissue remodeling (8, 18, 21), although the mechanisms underlying this phenotypic switch have not been fully elucidated.

## Macrophage Activation Markers

As discussed above, macrophages play a crucial role in both hepatic fibrogenesis and resolution of fibrosis, and several macrophage-released molecules are involved in these processes. Thus, plasma concentrations of such molecules may reflect the degree of fibrosis in liver diseases and help clinicians determine disease stage, prognosis, and response to intervention.

Scavenger receptors are expressed by macrophages as part of a large receptor panel accountable for regulation of endocytosis, phagocytosis, adhesion, and signaling. Some scavenger receptors are promising indicators of the inflammatory load in liver diseases and have a potential prognostic value (22). The most intensively studied proteins are cluster of differentiation 163 (CD163) and the mannose receptor (MR/CD206). CD163, a hemoglobin-haptoglobin scavenger receptor, is exclusively expressed by monocytes and macrophages (23, 24) and exerts its main biological function through elimination of hemoglobin-haptoglobin complexes during hemolysis (25). A soluble form (sCD163) is present in the plasma (26), and enzymatic receptor cleavage of CD163 by the TACE/ADAM17 system is highly upregulated in response to inflammatory stimuli, including the PAMP lipopolysaccharide (LPS) (27–30). The mannose receptor is primarily expressed by macrophages, dendritic cells and endothelial cells (31) and is involved in scavenging events including endogenous molecule clearance besides antigen presentation (32). Similar to sCD163, soluble mannose receptor (sMR) is present in plasma, and shedding by proteases is induced by PAMPs (Figure 1) (28, 33). Even though sCD163 and sMR share similarities regarding expression and stimuli leading to shedding, and their concentrations inter-correlate indicating concurrent shedding from activated macrophages, the biomarkers possess different immunogenic roles and may be differently regulated with fluctuations of serum concentrations in diverse immunological conditions (22). Furthermore, the very shedding intensity induced by various stimuli may differ between the two biomarkers (34).

## Macrophage Activation Markers in Liver Diseases

The dynamic processes of hepatic inflammation have been extensively studied over the last decades with increasing focus on macrophage activation markers and their utility in staging the degree of ongoing inflammation and fibrosis, as well as predicting disease outcome. In this review, when discussing non-alcoholic fatty liver disease (NAFLD), we will use the recently introduced and more appropriate consensus nomenclature of metabolic associated fatty liver disease (MAFLD) (35, 36). As previously summarized and illustrated in (22, 37, 38) plasma levels of sCD163 are moderately elevated in MAFLD (39), chronic hepatitis B virus (HBV) and chronic hepatitis C virus (HCV) infections (40), Wilson's disease (41, 42), and primary biliary cholangitis (43), and clearly reflects disease severity in MAFLD, HBV and HCV (39, 40, 44–47). In further support of a correlation between sCD163 concentrations and liver disease severity sCD163 levels show significant reductions after lifestyle interventions in MAFLD (48, 49) and after antiviral therapy in HBV and HCV (46, 50, 51). In manifest cirrhosis, sCD163 levels are even higher with a dramatic stepwise increase in parallel with Child-Pugh- and MELD-scores (52). Moreover, sCD163 values predict the degree of portal hypertension (53, 54) and are associated with variceal bleeding and prognosis (55, 56). In cirrhotic patients with hepatocellular carcinoma, sCD163 levels associate significantly with overall survival (57, 58). Hepatic expression of CD163 is significantly increased in patients with acute viral hepatitis compared with chronic viral hepatitis (59). Accordingly, the most prominent elevations of plasma sCD163 are seen in acute liver injury with intense inflammation including alcoholic hepatitis (60, 61), acute liver failure (62, 63), and acute-on-chronic liver failure (ACLF) (64), where it shows great potential as an independent predictor of short-term mortality.

Similarly, sMR has become of increasing interest as a marker of inflammation in liver disease. In children with MAFLD, sMR is elevated compared with non-overweight controls (65). sMR mimics sCD163 in chronic HBV and HCV, with increasing plasma concentrations in association with incrementing severity of hepatic inflammation (51, 66) and persistent reduction after antiviral therapies (51, 67). sMR is substantially elevated in patients with cirrhosis with significant correlation with Child-Pugh-score (56). Furthermore, it is a predictor of long-term survival and for the occurrence of cirrhosis-associated complications including decompensating events such as ascites, hepatic encephalopathy, and upper gastrointestinal hemorrhage due to portal hypertension within 1 year (56). Lastly, patients suffering from acute liver injury due to acetaminophen overdose, acute cirrhosis decompensation, ACLF, and alcoholic hepatitis have markedly increased plasma levels of sMR with 2.5- to 5-fold higher median values compared with healthy individuals (64, 68, 69). In addition, combining the CLIF-C ACLF score and sCD163 improves the prediction of 90 days mortality, while sMR in addition to the CLIF-C acute decompensation (AD) score improves the prediction of 90 and 180 days mortality (64, 70).

In summary, multiple studies have documented that plasma concentrations of the macrophage activation markers sMR and especially sCD163 are reliable indicators of ongoing hepatic inflammation, and potential useful tools to predict disease outcome including mortality.

## sCD163 and sMR in Liver Fibrosis

As outlined above, a pivotal event in the hepatic inflammatory response is activation of resident and recruited macrophages responsible for further signaling events and subsequently resulting in a spectrum of scenarios ranging from resolution of inflammation to maintenance or intensification of the response, activation of hepatic stellate cells with fibrosis development, and/or induction of fibrolysis. There is a solid foundation to consider sCD163 and sMR as important and clinically useful markers of inflammation in liver disease, but they may equally reflect fibrosis severity. The latter will be reviewed below with a specific focus on MAFLD and chronic HBV and HCV where the most robust data is available.

### MAFLD

The prevalence of obesity is increasing worldwide leading to a substantially increased health care burden related to diseases associated with obesity. This includes MAFLD and MAFLD with steatohepatitis with or without fibrosis (1, 35, 36). MAFLD is related to insulin resistance, obesity, type 2 diabetes mellitus (71); and suggested to be the hepatic manifestation of the metabolic syndrome (72, 73). Key immunomodulatory events comprise hepatic lipid accumulation and increased translocation of bacterial components from the gut. If the lipid load surpasses the hepatic metabolic capacity, the dysregulated hepatic lipid metabolism will result in toxic lipid intermediates. These intermediates will be recognized as DAMPs by liver macrophages and initiate activation through PRRs. Gut derived bacterial components (e.g., LPS) potentiates this process, further increasing liver inflammation (Figure 1). A recent study of 40 non-diabetic and mostly non-obese patients with biopsy-proven MAFLD and varying severity of hepatic steatosis and fibrosis, indicated a link between adipose tissue insulin resistance and Kupffer cell activation since sCD163 concentrations associated with circulating free fatty acids, lipolysis rate and insulin resistance in adipose tissue (74). Persistent and excessive inflammation entails fibrogenic events including activation of myofibroblasts, which are highly dependent on release of inflammatory and pro-fibrotic cytokines such as TGF-β from activated Kupffer cells and recruited macrophages (74–76). Even though fibrosis progresses slowly in MAFLD (77) ~20% are rapid progressors (78), and there is an urgent need for improved tools to non-invasively diagnose and evaluate liver fibrosis as well as to predict progression risks and treatment effects.

**Figure 2:**
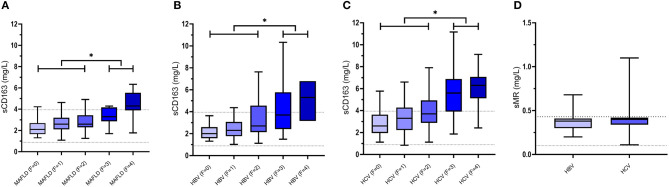
Concentrations of sCD163 in MAFLD **(A)**, chronic HBV infections **(B)** and chronic HCV infections **(C)** as well as concentrations of sMR in chronic HBV infections and chronic HCV infections **(D)** presented by means/medians, interquartile ranges as well as minimum and maximum values. In patients with MAFLD, chronic HBV infections and chronic HCV infections plasma levels of sCD163 increase with increasing fibrosis grade (F0-F4) (40, 44, 51, 67) with significantly higher levels in patients with fibrosis grades F3-F4 compared with patients with fibrosis grades F0-F2 (*p-value <0.001). Dotted lines represent upper and lower reference values for healthy controls (26, 34). MAFLD, metabolic associated fatty liver disease; HBV, hepatitis B virus; HCV, hepatitis C virus; sCD163, soluble cluster of differentiation 163; sMR, soluble mannose receptor.

In morbidly obese adults undergoing bariatric surgery and perioperative liver biopsy, sCD163 was significantly associated with the Kleiner fibrosis score. In addition, patients with a high fibrosis score had significantly higher preoperative sCD163 levels (39), an association confirmed in two independent cohorts of patients with biopsy-proven MAFLD (Figure 2A). sCD163 as a sole marker performed well in detecting advanced fibrosis, and the addition of sCD163 to the established and widely applied model NAFLD Fibrosis Score (NAFLD-FS) improved the predictive capacity of the latter (44). sCD163 and NAFLD-FS had comparable sensitivities, 84 and 80%, respectively, for predicting advanced fibrosis (F ≥ 3) using low cut-off values in an Australian cohort. However, in an Italian cohort the sensitivities of sCD163 and NAFLD-FS for predicting advanced fibrosis were much lower (~40%), though still comparable (44). Another research group reported a similar significant association between sCD163 and hepatic fibrosis in obese individuals undergoing bariatric surgery (45). Recently, results from 40 patients with MAFLD and available liver histology and sCD163 quantifications as well as a comprehensive metabolic assessment clearly demonstrated an association between sCD163 and the stage of fibrosis (74).

In obese children undergoing lifestyle interventions, sCD163 was significantly higher at baseline compared with 1-year follow-up in children with a high level of a non-invasive surrogate measure for fibrosis [the Pediatric NAFLD Fibrosis Index (79)]. Following lifestyle intervention, sCD163 levels decreased in association with improvements in metabolic dysfunction (48). However, a recent study of sCD163 and sMR in children with MAFLD reported no significant associations between either sCD163 or sMR and fibrosis assessed from liver biopsies, which was in clear contrast with the results shown in adult cohorts and may suggest different mechanisms in terms of macrophage activation and fibrosis development in children compared with adults with MAFLD (65).

### HBV and HCV

Chronic infection with HBV and HCV is maintained by continued release of viral components by infected cells (80, 81), thus interacting with the intrahepatic innate immune system including Kupffer cells and hepatic stellate cells (Figure 1). These are directly activated by HBV and HCV components in addition to an indirect stimulation by differentiated pro-fibrogenic liver macrophages (82, 83). Genetic studies have recently elucidated an association between sCD163 levels and expression of specific gene variants known to promote hepatic inflammation and fibrogenesis in patients with chronic HCV, thus supporting a pivotal role for Kupffer cell activation in the development and progression of hepatic fibrosis (14, 84). Several studies have measured sCD163 and sMR in chronically HBV and HCV infected individuals and related biomarker concentrations to fibrosis scores. In a small population of HCV patients assessed by the non-invasive FibroScan® sCD163 was associated with liver fibrosis (66). Data concerning liver histology and biochemistry from a large treatment-naïve cohort of 513 patients with chronic HCV and 200 patients with chronic HBV were published in 2014 (40). In general, HCV patients had higher sCD163 concentrations for the same fibrosis score as HBV patients. In both HCV and HBV, sCD163 increased in association with histological stages of fibrosis (Figures 2B,C). Supporting a direct association between sCD163 and fibrosis in HCV patients a CD163-HCV-fibrosis-score was superior to the common fibrosis scoring tools, APRI and FIB-4, for predicting significant fibrosis (40). The sCD163-based HCV fibrosis score has been successfully validated (50). A CD163-HBV-fibrosis-score was also presented and evaluated, but was not significantly superior to APRI and FIB-4 (40). Dultz et al. showed that chronic HBV patients with an Ishak fibrosis score ≥2 had significantly higher levels of plasma sCD163 than F0-F1 patients. Thus, sCD163 may be a useful biomarker to discriminate chronic HBV treatment-naïve patients with minimal fibrosis from patients with significant fibrosis (46). Recently, sCD163 and sMR were measured in a cohort of chronic HBV patients before and after nucleoside-analogue treatment (Figure 2D). The two macrophage activation markers showed a weak association with the Ishak fibrosis score (51). In this study, the CD163-HBV-fibrosis-score was validated and performed similarly to APRI and FIB-4 for prediction of significant fibrosis (51). A similar study of sCD163 and sMR in chronic HCV patients reported significant reductions of liver stiffness evaluated by transient elastography (FibroScan® or ARFI scan®) following antiviral treatment (Figure 2D). Concentrations of sCD163 and sMR correlated with liver stiffness at baseline and follow-up, though no consistent conclusion on the predictive value of the markers in relation to fibrosis severity was presented (67). Hence, the high baseline concentrations of sCD163 and sMR may not solely reflect fibrosis, since significantly higher viral load and alanine aminotransferase were present at baseline, indicating a considerable component of active liver inflammation.

In summary, there is a well-documented association between plasma concentrations of sCD163 and hepatic fibrosis in patients with chronic HBV and HCV, with a decline after antiviral treatment. The results for HCV are the most concordant.

## Concluding Remarks

Plasma levels of sCD163 and sMR may be clinically useful as they directly reflect macrophage activation in liver diseases. Since macrophages play a significant immunomodulatory role in acute and chronic inflammatory liver disease, including an interplay with hepatic stellate cells, sCD163 and sMR have gained interest as potential markers of hepatic inflammation and fibrosis. Results from several studies in humans indicate usefulness of sCD163 and sMR in estimating the degree of ongoing inflammation in acute and chronic liver diseases in general and in assessing fibrosis severity in patients with MAFLD as well as chronic HBV and HCV infections. Since hepatic inflammation and fibrosis are inter-related events—both being consequences of the continuum of immunological activities in chronic liver diseases—the inflammatory burden must be considered as a possible limitation to the use of sCD163 and sMR as biomarkers of liver fibrosis. Moreover, sCD163 may be elevated in other diseases involving activation of monocytes and macrophages, including Gaucher disease (characterized by excessive macrophage proliferation), hemophagocytic syndrome, infectious diseases, chronic inflammatory diseases, and leukemia, and may be markedly elevated in septic patients (85), limiting its clinical utility. Pulmonary fibrosis may also result in elevations of circulating sCD163 (86). Hence, critical and thorough evaluation of the clinical setting is important before sCD163 and sMR are measured with the purpose of assessing liver fibrosis. However, in the urgent need for improved non-invasive disease scoring tools, especially concerning MAFLD and chronic viral hepatitis B and C, sMR and sCD163 possess great potentials toward being included in fibrosis assessments and may even reflect treatment effects. Further, from a therapeutic perspective, the membrane bound CD163 and perhaps MR may hold promise as entry molecules for liver macrophage-targeted drug delivery (87, 88). An advantage of CD163 is rapid internalization of ligands limiting systemic drug exposure (89) as well as providing a directed action of the applied drug (90). Consequently, intravenous injections of CD163-directed anti-IgG-dexamethasone conjugate in rats on a high fructose diet significantly reduced hepatic inflammation and fibrosis (88). However, future studies in humans are needed to further elucidate the therapeutic potential of macrophage receptors, thereby possibly extending the treatment options for patients with chronic liver diseases.

## Author Contributions

RG and HG: conceptualization. RG: writing–original draft. RG, MK, TL, KK, JG, HM, and HG: writing–review and editing. All authors approved the final version of the manuscript.

## Conflict of Interest

The authors declare that the research was conducted in the absence of any commercial or financial relationships that could be construed as a potential conflict of interest.
